# DNA functionalization by dynamic chemistry

**DOI:** 10.3762/bjoc.12.203

**Published:** 2016-10-06

**Authors:** Zeynep Kanlidere, Oleg Jochim, Marta Cal, Ulf Diederichsen

**Affiliations:** 1 Institute of Organic and Biomolecular Chemistry, Georg-August University Göttingen, Tammannstrasse 2, D-37077 Göttingen, Germany

**Keywords:** base-pairing, base-pair mismatch, DNA functionalization, DNA templates, dynamic combinatorial chemistry, D-threoninol based scaffolds

## Abstract

Dynamic combinatorial chemistry (DCC) is an attractive method to efficiently generate libraries of molecules from simpler building blocks by reversible reactions under thermodynamic control. Here we focus on the chemical modification of DNA oligonucleotides with acyclic diol linkers and demonstrate their potential for the deoxyribonucleic acid functionalization and generation of libraries of reversibly interconverting building blocks. The syntheses of phosphoramidite building blocks derived from D-threoninol are presented in two variants with protected amino or thiol groups. The threoninol building blocks were successfully incorporated via automated solid-phase synthesis into 13mer oligonucleotides. The amino group containing phosphoramidite was used together with complementary single-strand DNA templates that influenced the Watson–Crick base-pairing equilibrium in the mixture with a set of aldehyde modified nucleobases. A significant fraction of all possible base-pair mismatches was obtained, whereas, the highest selectivity (over 80%) was found for the guanine aldehyde templated by the complementary cytosine containing DNA. The elevated occurrence of mismatches can be explained by increased backbone plasticity derived from the linear threoninol building block as a cyclic deoxyribose analogue.

## Introduction

The well-defined duplex structure, self-assembling by base-pair recognition, and the accessibility by solid-phase synthesis make DNA oligonucleotides an ideal supramolecular scaffold in a wide field of applications [1–2]. In recent years oligonucleotides especially were applied to self-assembly into artificial nanostructures [3–9]. Preparation of oligonucleotides for new applications requires the introduction of additional functional groups into its native structure [10–11]. Such chemically modified oligonucleotides are useful intermediates for their subsequent functionalization through post-synthetic protocols [11–13]. Within a post-synthetic strategy, a nucleotide analog is modified with a reactive functional group, incorporated into oligonucleotides by standard solid–phase synthesis and reacted with the desired molecules on the oligonucleotide level. As amines and thiols are among the widely used groups introduced for the post-synthetic modifications, the acyclic threoninol linker (2-amino-1,3-butanediol) [14–21] constitutes an attractive choice for oligonucleotide functionalization. Threoninol can be introduced in oligonucleotides via the corresponding phosphoramidite generating a ribose-free abasic site on the backbone that provides the amine group for later functionalization [22–28]. Similarly, a thiol functionality can be introduced by substitution of the amine group of threoninol and incorporated into the oligonucleotide backbone. The amine and thiol groups can be used for further oligonucleotide functionalization reacting these sites with functional molecules like metal ligands or fluorophores. Functional molecules of interest can be tethered post-synthetically in an irreversible manner as amide or reversibly as imine or thioester.

Recent advances in dynamic combinatorial chemistry [29–40] have enabled the utilization of presynthesized oligomers with abasic sites on the backbone for the addition of individual monomeric nucleobases and consider the synthesis of new oligonucleotide analogues possessing different backbone topologies [41]. Ghadiri et al. employed this approach for an enzyme-free synthesis of an oligonucleotide analogue with a peptide backbone carrying nucleobases on its amino acid side chains [42] while Bradley et al. used the backbone of a peptide nucleic acid (PNA) with abasic sites which gives a reactive secondary amine for reversible attachment of aldehyde modified nucleobases [43]. Moreover, the DNA template-directed selection of one nucleobase from the reaction mixture with the amine or thiol functional group was investigated [44–47].

**Figure 1:**
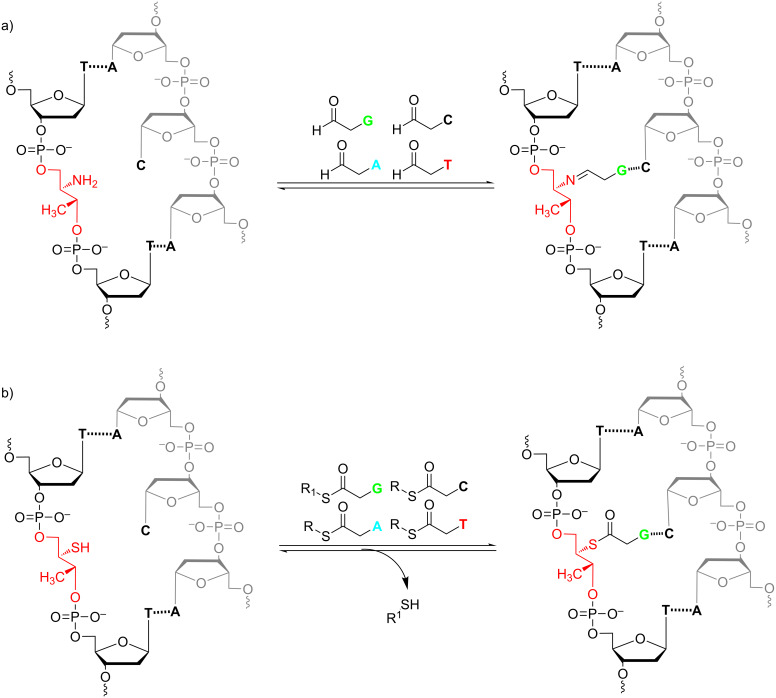
Template-directed dynamic chemistry assay for the attachment of modified nucleobase monomers to an abasic backbone. a) Reversible imine exchange reaction. b) Reversible thioester exchange reaction.

In our studies, dynamic chemistry is applied for post-synthetic functionalization of the threoninol based modified oligonucleotides in a reversible manner. Here we synthesized the phosphoramidite building blocks derived from D-threoninol which contain protected amine or thiol groups. These building blocks are used for later incorporation into oligonucleotides via solid-phase synthesis. Using these modified oligonucleotides and single strand DNA templates, we generated the libraries of reversibly interconverting building blocks – dynamic combinatorial libraries (DCL) (Figure 1). The abasic strand and its complementary template strand are spontaneously assembled into a double helix through Watson–Crick base-pairing and the incoming nucleobase monomer benefits from the hydrogen bonding recognition by the respective nucleobase in the template strand. The reversible attachment generates a dynamic system that enables the combinatorial screening of the best bound nucleobase by allowing a rapid and continuous exchange between the threoninol site and the set of nucleobase monomers.

In case of an amine group on the backbone, a reversible imine exchange reaction with aldehyde modified nucleobases was performed (Figure 1a). In the presence of a thiol group on the backbone, a thioester exchange reaction with thioester modified nucleobases was expected (Figure 1b).

## Results and Discussion

### D-Threoninol-based building blocks: design and synthesis

**Scheme 1:**
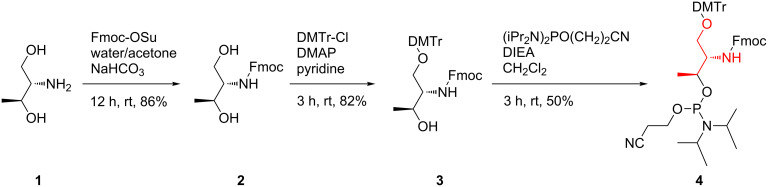
Synthesis of the phosphoramidite building block **4** [23]. DMTr: dimethoxytrityl group, Fmoc: 9-fluorenylmethylcarbonyl, DMAP: 4-dimethylaminopyridine, DIEA: *N,N*-diisopropylethylamine.

Two D-threoninol-based phosphoramidite building blocks containing orthogonally protected amine **4** or thiol **11** moieties were successfully synthesized. As presented in Scheme 1 phosphoramidite **4** was obtained according to the procedures previously described in literature [14,23].

**Scheme 2:**
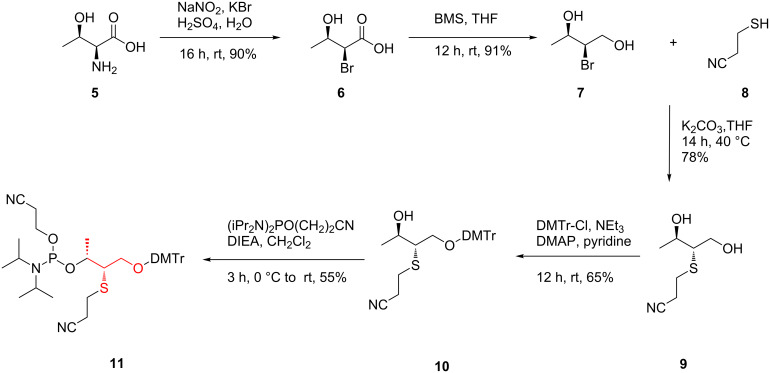
Synthesis of the phosphoramidite building block **11**.

In order to obtain phosphoramidite **11** we have developed a synthesis (Scheme 2) based on L-threonine as a starting material. L-Threonine was converted to bromo-derivative **6** by a diazotization reaction using sodium nitrite followed by potassium bromide substitution under overall retention of configuration due to double inversion. Next, the subsequent reduction of carboxylic acid **6** to alcohol **7** was achieved by borane dimethyl sulfide (BMS) under dry conditions. The reaction between alcohol **7** and 3-mercaptopropanenitrile (**8**) resulted in substitution of bromine to the thiol group and finally the introduction of the thiol functional group. The 3-mercaptopropanenitrile (**8**) was synthesized separately in two steps from acrylonitrile or the 3-chloropropanenitrile according to the previously described procedure [48–50]. Next, the obtained compound **9** containing two hydroxy groups and the cyanoethyl protected thiol group was converted into the phosphoramidite being compatible with conditions of solid-phase oligonucleotide synthesis. The DMTr protecting group was incorporated and the conversion of the secondary alcohol **10** to phosphoramidite **11** was performed. The base-labile cyanoethyl group [51–52] is known to be resistant under synthesis conditions for the preparation of the phosphoramidite building block and for solid-phase oligonucleotide synthesis [49,53].

### Building blocks compatibility with solid-phase synthesis of DNA single strands

Phosphoramidites **4** and **11** were introduced at position seven of 13mer oligonucleotides **ON1** and **ON2** applying automated solid-phase synthesis (Table 1 and Supporting Information File 1, Table S1). The last step in the oligonucleotide synthesis involved the deprotection of the amine group using ammonium hydroxide at 55 °C. The Fmoc protecting group of oligonucleotide **ON1** was removed, however, the cyanoethyl group as a base-labile protecting group of the thiol was not removed quantitatively from the oligonucleotide **ON2** [54–58].

**Table 1:** Sequences of modified oligonucleotide and templates used in this work.

Strand	Sequence (5’-3’)

**ON1**	CGCTAT**X**TATCGC^a^
**ON2**	GCGATA**Y**ATAGCG^a^
**T_C_**^b^	GCGATA**C**ATAGCGGTT^c^
**T_G_**^b^	GCGATA**G**ATAGCGGTT
**T_T_**^b^	GCGATA**T**ATAGCGGTT
**T_A_**^b^	GCGATA**A**ATAGCGGTT

^a^**X** and **Y** represent the abasic site with amino or thiol group, respectively. ^b^Here, the capital letter index of **T** stands for the cytosine, guanine, thymine or adenine nucleobase positioning opposite to the amine on **ON1**. ^c^Nucleobases that are opposite to the abasic site of the modified strand are shown in bold letters. The GTT sequence at the 3’-terminus was added to facilitate separation by HPLC.

### Dynamic template-driven assembly of double strand libraries

**Figure 2:**
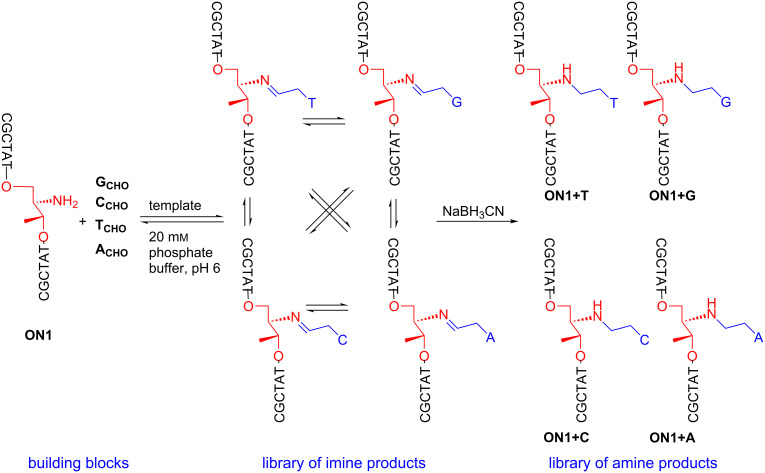
Initial building blocks of a dynamic combinatorial library, a library of all possible imine products and a library of amine products after reduction.

**Figure 3:**
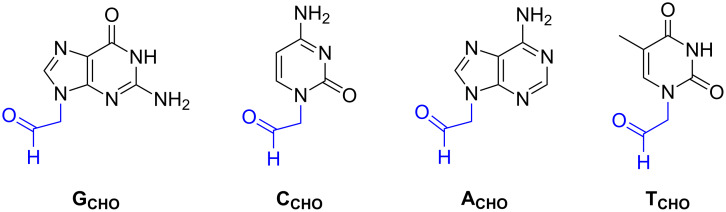
Set of aldehyde-modified nucleobases used in dynamic chemistry assay.

Oligonucleotide **ON1** was used for further investigations towards dynamic libraries of double strand DNA constructs. Oligonucleotide **ON1** was used with the deprotected amine group in the reaction of forming the imine bond between **ON1** and four nucleobase aldehydes (**G_CHO_**, **C_CHO_**, **A_CHO_**, **T_CHO_**) in the presence of complementary template strands **T_C_**, **T_G_**, **T_T_** or **T_A_** (Table 1). The respective template strand should control the incorporation of the corresponding nucleobase reversibly linked as imine (Figure 2). The structures of four nucleobase aldehydes were shown in Figure 3. These compounds were synthesized according to the procedures described previously [59–61].

At the beginning we determined the conversion of **ON1** with the respective nucleobase aldehydes in presence of the complementary DNA template strand (Table 1). The **ON1** oligonucleotide was allowed to react with only one nucleobase in order to produce the individual product (Supporting Information File 1, Figure S1, Table S2). A reaction between **G_CHO_** and **ON1** in the presence of **T_C_** was accomplished with complete conversion of **ON1** into the guanine incorporated product (**ON1+G**). The reactions with cytosine (**C_CHO_**) as well as adenine (**A_CHO_**) aldehydes gave similar yields, however, with lower conversion level of **ON1** compared to the previous case. Finally, lowest conversion was observed for the reaction of **ON1** with the **T_A_** template and the thymine aldehyde (**T_CHO_**) (Supporting Information File 1, Table S3).

### The composition of libraries: base-pairing selectivity

To determine the influence of the DNA template on nucleobase incorporation into the strands at abasic site through imine attachment to the amine group, four reactions were carried out under identical conditions (pH 6, 20 mM phosphate buffer), but differing in the applied template strand. The 13mer oligonucleotide **ON1** was mixed with one of the four complementary template strands (**T_C_**, **T_G_**, **T_T_** or **T_A_**, Table 1) in a 1:1 molar ratio. All four nucleobase aldehydes (**G_CHO_**, **C_CHO_**, **A_CHO_**, **T_CHO_**) were added in excess amount and in equimolar concentrations.

**Figure 4:**
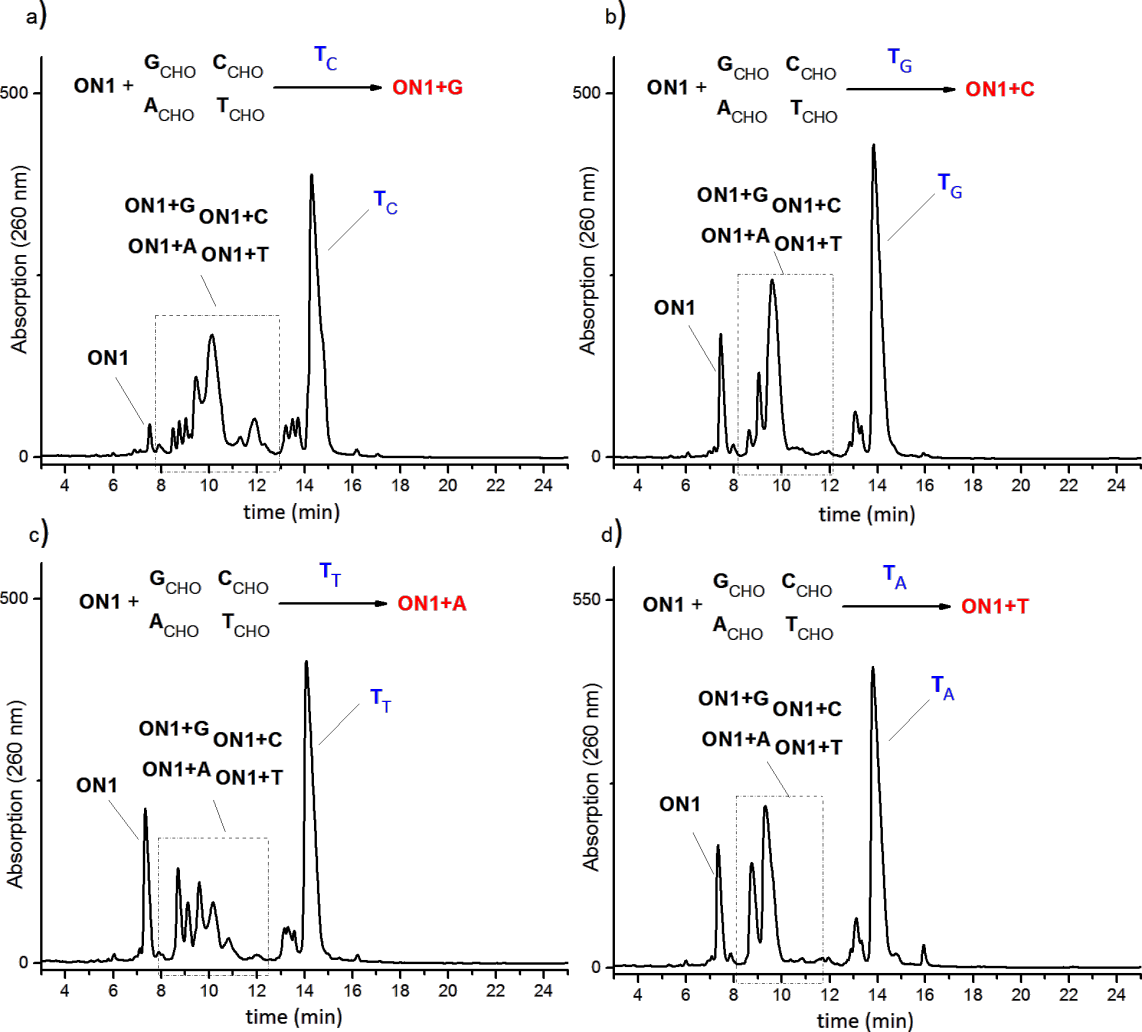
Representative HPLC chromatograms for the mixture containing **ON1** and four nucleobases in the presence of a DNA-template: a) **T_C_**; b) **T_G_**; c) **T_T_**; d) **T_A_**. The chromatograms were performed after reductive amination of the samples (for more details see Supporting Information File 1).

**Figure 5:**
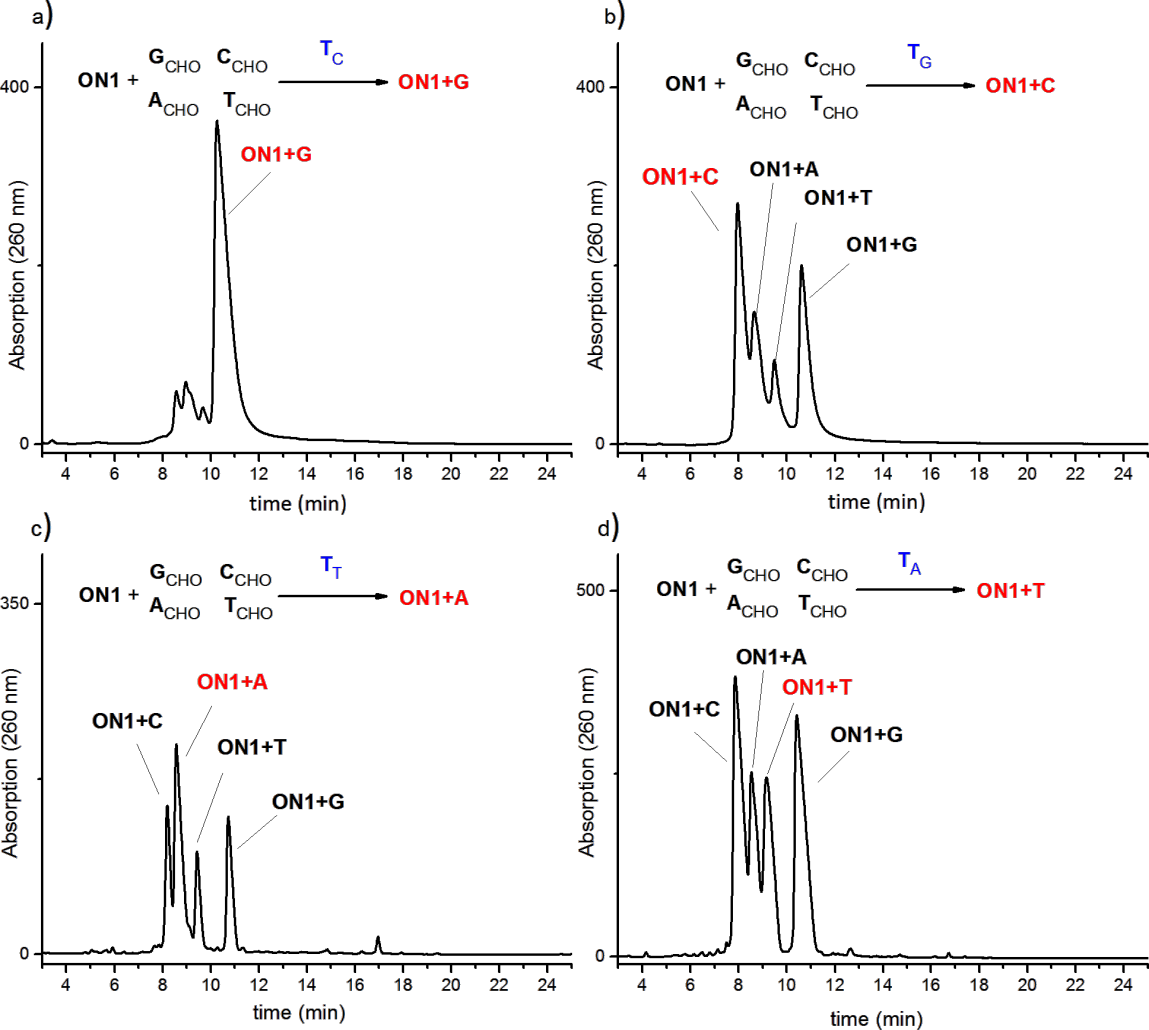
Representative HPLC chromatograms obtained using elevated pH, for samples collected from first purification step (in Figure 4 marked with dashed line). The samples were collected from the reaction of **ON1** and four nucleobases in the presence of a DNA-template: a) **T_C_**; b) **T_G_**; c) **T_T_**; d) **T_A_**. The chromatograms were obtained after reductive amination of the samples (for more details see Supporting Information File 1).

Sodium cyanoborohydride (NaBH_3_CN) was used for irreversible conversion of the imine products obtained in equilibrium into respective amines (Figure 2), thereby enabling the isolation and analysis of the library derived from oligonucleotide **ON1**. Anion exchange high-performance chromatography was used for the analysis of the final reaction mixture. The reaction mixtures were composed of six oligonucleotides: the unreacted initial strand **ON1**, one of its complementary strands **T_C_**, **T_G_**, **T_T_** or **T_A_** and the four possible product strands **ON1+G**, **ON1+C**, **ON1+A**, **ON1+T** (Figure 4). HPLC separation of the four possible products in the same reaction mixture was challenging because the lengths of the starting sequence CGCTAT**X**TATCGC (**ON1**) and the product sequences (**ON1+G**, **ON1+C**, **ON1+A**, **ON1+T**) were identical differing only by one nucleobase in the central position **X**. As shown in Figure 4 all possible products were eluted as a mixture separated by anion exchange HPLC at 80 °C; under these conditions dissociation of obtained oligonucleotide double strands is provided. Well separated signals correspond to the temple strands and starting oligonucleotide **ON1**. The obtained four new strands (**ON1+C, ON1+G, ON1+T, ON1+A**) were eluted with similar retention time and broad elution profiles. Therefore, the product containing fractions were subjected to a second HPLC purification step applying basic conditions (pH 12) to separate these compounds (Figure 5). At high pH deprotonation of guanine and thymine allow better separation. As indicated by the elution profiles in Figure 5, the template strands significantly affect the composition of the dynamic library. The control experiment lacking the template provided a nearly equal distribution of oligonucleotides (results are not given here).

The highest selectivity of more than 80% was obtained for the incorporation of **G_CHO_** (Figure 5a) with the complementary template strand (**T_C_**). The Watson–Crick base-pairing with three hydrogen bonds together with a high-stacking contribution of purine nucleobases seems to be beneficial. The selectivity for the incorporation of the other aldehydes is significantly lower (20–40%). Especially for **C_CHO_** with the complementary guanine (**T_G_**) template nucleobase incorporation was not supported by Watson–Crick **G·C** base-pairing (Figure 5b). The templating reactions were repeated four times applying different HPLC conditions. In all cases, incorporation of **G_CHO_** in the presence of template **T_C_** was obtained with clear preference.

In case of incorporation of the thymine aldehyde the **T_A_** containing template was not effective by supporting the expected **ON1+T** product as it would have been supported by the **A·T** base pair formation (Figure 5d). Moreover, this dynamic library is even dominated by the two **A·C** and **A·G** mismatches indicating a highly flexible arrangement of the incoming nucleobases. In general, the low template directed selectivity for incorporation of individual nucleobases is likely due to the higher flexibility in the backbone derived from threoninol units. The canonical Watson–Crick **A·T** and **C·G** base pairs are most energetically favorable, while other purine–purine (like **A·A**, **G·G**) mismatches are less frequent than **T·G** and **C·A** ones. These results indicate that the selectivity of base pairing is not only driven by the number and strength of hydrogen bonds formed between two bases, but also by the backbone plasticity providing the frame for this interaction.

## Conclusion

The efficient synthesis and DNA incorporation of two D-threoninol based phosphoramidite building blocks with orthogonally protected amine or thiol functional groups was described. Therefore, DNA analogues were presented that can be covalently functionalized by imine or thioester formation. In principle this concept allows dynamic DNA functionalization with all kind of functional or recognition units at positions that were modified with the threoninol deoxyribose analogous by solid phase synthesis. As proof of principle the 13mer oligonucleotide containing a threoninol derived amine functionality was submitted to dynamic combinatorial library (DCL) studies for DNA template directed nucleobase incorporation. Whereas a significant preference for the incorporation of the guanine unit directed by a complementary cytosine was found, linkage of the other nucleobases was not at all selective and it seems likely that the high flexibility of the threoninol as a deoxyribose analogue does not allow better selection. Due to difficulties in deprotection of the thiol group, oligonucleotides with threoninol derived thiol functionality are still under investigation as well as the simultaneous functionalization of DNA oligonucleotides at various positions with different kind of functional units.

## Supporting Information

File 1 Experimental procedures and NMR spectra of compounds **6**, **7**, **9–11** as well as preparation and analytical data of oligonucleotides **ON1**, **ON2**, **ON1+G**, **ON1+C**, **ON1+A** and **ON1+T**.
