# Supplementary material for: DNA functionalization by dynamic chemistry
Source: Beilstein J Org Chem. 2016 Oct 6;12:2136–44. doi: 10.3762/bjoc.12.203 (PMC5082583; doi:10.3762/bjoc.12.203)
Supplement: File 1 — Experimental procedures and NMR spectra of compounds 6, 7, 9–11 as well as preparation and analytical data of oligonucleotides ON1, ON2, ON1+G, ON1+C, ON1+A and ON1+T. [file Beilstein_J_Org_Chem-12-2136-s001.pdf]

Supporting Information

for

DNA functionalization by dynamic chemistry

Zeynep Kanlidere[1], Oleg Jochim[1], Marta Cal[1] and Ulf Diederichsen*[1]

Address: [1]Institute of Organic and Biomolecular Chemistry, Georg-August University Göttingen, Tammannstrasse 2, D-37077 Göttingen, Germany

Email: Ulf Diederichsen - udieder@gwdg.de

* Corresponding author

Experimental procedures and NMR spectra of compounds 6, 7, 9–11 as well as preparation and analytical data of oligonucleotides ON1, ON2, ON1+G, ON1+C, ON1+A and ON1+T

Table of Contents

Experimental procedures and analytical data of compounds **6**, **7**, **9-11** **S2**

Synthesis and analytical data of oligonucleotides **ON1** and **ON2** **S5**

Experimental procedures for dynamic imine exchange reactions **S6**

Analytical data of products in dynamic library **S7**

Experimental procedures and analytical data of compounds 6, 7, 9–11

General methods

Compounds **2**, **3**, **4** [1,2] and **8** [3] were prepared according to published procedures. All chemical reagents were purchased from standard suppliers. Dry solvents were supplied from Acros Organics, Sigma Aldrich or Fluka and were stored over molecular sieves (4 Å). All moisture and air sensitive reactions were carried out in oven dried glassware under an inert argon atmosphere. Analytical TLC was performed on Merck TLC aluminium-backed silica gel 60 F_{254} plates (layer thickness: 0.20 mm). Visualization of the spots was carried out using UV light (254 nm) and/or staining with a ninhydrine solution (3.0 g ninhydrine in 100 mL ethanol). Aldehyde and acetal products were visualized using 2,4-dinitrophenylhydrazine solution (60 mL H_2SO_4, 80 mL H_2O, 200 mL 95% ethanol). Column chromatography was carried out on Merck silica gel 60 (0.040–0.063 mm, 230–400 mesh size) under flash conditions. Solvents for flash chromatography (ethyl acetate, pentane, methanol, dichloromethane) were distilled prior to use. ^{1}H-, ^{13}C- and ^{31}P- NMR spectra were recorded on a Varian Unity 300 spectrometer, a Varian Inova 500 or 600, a Bruker 300 or 400 spectrometers. All spectra were recorded at room temperature and were referenced internally to solvent reference frequencies. For the calibration of ^{31}P- NMR signals, 85% phosphoric acid was used as an external standard. Chemical shifts (δ) are reported in ppm and coupling constants (*J*) are given in Hertz (Hz). ^{13}C-spectra were recorded as broadband decoupled ATP spectra. Data from [^{1}H,^{1}H]-COSY, HSQC and HMBC experiments were used to interpret the signals. Electrospray ionization-mass spectra (ESI-MS) were obtained with a Finnigan instrument (type LGC or TSQ 7000) or Bruker spectrometers (Apex-Q IV 7T and micrOTOF API). High Resolution (HR) spectra were obtained with the Bruker Apex-Q IV 7T or the Bruker micrOTOF, respectively. For ESIMS in the negative mode, solutions of the compounds in acetonitrile were used.

Analytical data:

(2*S*,3*R*)-2-Bromo-3-hydroxybutanoic acid (6) [4]

L-Threonine (25.0 g, 210 mmol) and potassium bromide (87.4 g, 73.4 mmol) were dissolved in sulfuric acid (2.5 M, 500 mL) followed by cooling it down to 0 °C under stirring. A mixture of sodium nitrite (22.0 g, 32.8 mmol) in water (100 mL) was added dropwise over 3 h at 0 °C. The obtained solution was stirred for 12 h at room temperature and then extracted with Et_2O (3 × 250 mL). The combined organic layers were washed with saturated brine (2 × 250 mL),

dried over $MgSO_4$, filtered and evaporated under reduced pressure to obtain the product **6** as a yellow oil.

Yield: 90% (34.4 g).

^{1}H NMR (300 MHz, $CDCl_3$): δ = 1.25 (d, $^3J_{H\text{-}H}$ = 6.2 Hz, 3H, CH_3), 4.15 (q, $^3J_{H\text{-}H}$ = 6.2 Hz, 1H, CH), 4.22 (d, $^3J_{H\text{-}H}$ = 3.2 Hz, 1H, CH), 7.25 (s_{br}, 1H, OH) ppm. **^{13}C-NMR** (75 MHz, $CDCl_3$, RT): δ = 19.8 (1C, CH_3), 52.5 (1C, CH), 67.5 (1C, CH), 71.9 (1C, CO_2H) ppm.

ESI-MS *m/z* = 184.9 $[M+H]^+$, 206.9 $[M+Na]^+$. **HR-MS (ESI)**: calcd. *m/z* = 184.9631 for $C_4H_8O_3Br$, found 184.9637.

(2*S*, 3*R*)-2-Bromobutane-1,3-diol (7) [4]

Compound **6** (15 g, 81.9 mmol) was dissolved in dry THF (350 mL) under argon atmosphere. A 10 M solution of BMS (19.2 ml, 204 mmol) in dry THF (50 mL) was added dropwise for 1 h at 0 °C. The mixture was stirred for additional 12 h at room temperature and quenched with water (20 mL) upon cooling. The resultant solution was extracted with EtOAc (3 x 250 mL). The combined organic layers were washed with saturated brine (250 mL), dried over $MgSO_4$, filtered and the solvents were evaporated under reduced pressure. The crude product **7** was an oil.

Yield: 91% (12.6 g)

^{1}H-NMR (300 MHz, $CDCl_3$): δ = 1.25 (d, $^3J_{H\text{-}H}$ = 6.2 Hz, 3H, CH_3), 2.41 (s_{br}, 2H, 2 x OH), 4.05-4.18 (m, 1H, CH), 4.22-4.40 (m, 3H, CH, CH_2) ppm. **^{13}C-NMR** (75 MHz, $CDCl_3$, RT): δ = 21.1 (1C, CH_3), 52.9 (1C, CH), 60.4 (1C, CH_2), 69.9 (1C, CH) ppm.

ESI-MS *m/z* = 190.9 $[M+Na]^+$. **HR-MS (ESI)**: calcd *m/z* = 190.9678 for $C_4H_9O_2BrNa$, found 190.9680.

3-(((2*S*, 3*R*)-1,3-Dihydroxybutan-2-yl)thio)propanenitrile (9)

To a degassed solution of 3-mercaptopropionitrile (3.00 g, 34.4 mmol) in dry DMF (250 mL) the potassium carbonate (4.46 g, 34.4 mmol) was added under argon atmosphere. The bromobutanediol **8** (4.47 g, 26.4 mmol) was added dropwise and the solution was heated up to 40 °C. The obtained mixture was stirred for additional 12 h at 40 °C. The solution was filtered at room temperature. The solvent was concentrated under reduced pressure and then extracted with EtOAc (3 × 250 mL). The combined organic layers were dried over $MgSO_4$ and the solvent was removed under reduced pressure. The crude product was purified by flash column chromatography using a mixture of pentane/ethyl acetate (5:1). Obtain product **9** was yellow oil.

Yield: 78% (3.61 g).

***R*f** = 0.35 (pentane/ethyl acetate, 5:1)

^{1}H-NMR (300 MHz, $CDCl_3$,): δ = 1.31 (d, $^{3}J_{H\text{-}H}$ = 6.2 Hz, 3H, CH_3), 2.45 (s_{br}, 2H, 2 x OH), 2.71 (t, $^{3}J_{H\text{-}H}$ = 7.0 Hz, 2H, CH_2), 2.88 (t, $^{3}J_{H\text{-}H}$ = 7.0 Hz, 2H, CH_2), 3.90-3.99 (m, 3 H, CH, CH_2), 4.08-4.22 (m, 1H, CH) ppm. **^{13}C-NMR** (75 MHz, $CDCl_3$, RT): δ = 18.3 (1C, CH_3), 18.7 (1C, CH_2), 27.6 (1C, CH_2), 43.9 (1C, CH), 64.7 (1C, CH_2), 73.8 (1C, CH), 118.3 (1C, CN) ppm.

ESI-MS *m/z* = 198.1 $[M+Na]^+$. **HRMS (ESI)**: calcd *m/z* = 198.0559 for $C_7H_{13}NO_2SNa$, found 198.0559.

3-(((2*S*,3*R*)-1-(Bis(4-methoxyphenyl)(phenyl)methoxy)-3-hydroxybutan-2-yl)thio)-propanenitrile (10)

Compound **9** (1.00 g, 5.71 mmol) was coevaporated three times with pyridine to remove any associated water and then the flask was filled with argon. To this solution, triethylamine (0.75 g, 7.42 mmol) and 2-(dimethylamino) pyridine (34.8 mg, 0.29 mmol) in dry pyridine (50 mL) were added. After the addition of the dimethoxytritylchloride (2.12 g, 6.28 mmol), reaction mixture was allowed to stir for 12 h at ambient temperature. The reaction solution was concentrated and the obtained oil was purified by flash column chromatography using a mixture of pentane/ethyl acetate (8:1 + 0.1% triethylamine) to obtain the compound **10**.

Yield: 65% (1.77 g). ***R*f** 0.25 (pentane/ethyl acetate, 8:1);

^{1}H-NMR (300 MHz, $CDCl_3$): δ = 1.25 (d, $^{3}J_{H\text{-}H}$ = 7.7 Hz, 3H, CH_3), 2.52-2.64 (m, 1H, CH), 2.74 (t, $^{3}J_{H\text{-}H}$ = 6.8 Hz, 2H, CH_2CN), 2.84 (t, $^{3}J_{H\text{-}H}$ = 6.8 Hz, 2H, CH_2CN), 3.60-3.69 (m, 3H, CH, CH_2), 3.79 (s, 6H, 2 x OCH_3), 6.83 (d, $^{3}J_{H\text{-}H}$ = 8.8 Hz, 4H, H_{Ar}), 7.31-7.38 (m, 5H, H_{Ar}), 7.46 (d, $^{3}J_{H\text{-}H}$ = 8.8 Hz, 4H, H_{Ar}) ppm. **^{13}C-NMR** (75 MHz, $CDCl_3$, RT): δ = 18.3 (1C, CH_3), 18.7 (1C, CH_2), 27.6 (1C, CH_2), 43.9 (1C, CH), 55.1 (2C, 2 x OCH_3), 64.7 (1C, CH_2), 73.8 (1C, CH), 85.8 (1C, C_{DMT}), 112.1 (4C, 4 x CH, C_{DMT}), 118.2 (1C, CN), 126.8, 127.2, 128.7, 129.6 (9C, 9 x CH, C_{DMT}), 139.2 (2C, C_{DMT}), 147.9 (1C, C_{DMT}), 158.2 (2C, C_{DMT}) ppm.

ESI-MS *m/z* = 500.2 $[M+Na]^+$. **HRMS (ESI)**: calcd *m/z* = 500.1866 for $C_{28}H_{31}NO_4SNa$, found 500.1854.

(3*S*)-4-(Bis(4-methoxyphenyl)(phenyl)methoxy)-3-((2-cyanoethyl)thio)butan-2-yl-(2-cyanoethyl) diisopropylphosphoramidite (11)

Compound **10** (1.00 g, 2.12 mmol) was coevaporated three times with pyridine and then dissolved in dichlormethane (50 mL). To this solution, redistilled diisopropylethylamine

(0.36 g, 2.76 mmol) and *N,N*-diisopropyl-2-cyanoethyl-phosphoramidite chloride (500 mg, 2.12 mmol) were added at 0 °C under argon atmosphere. The reaction mixture was allowed to stir for 3 h at room temperature. Next the mixture was diluted with dichlormethane and washed with saturated $NaHCO_3$. The aqueous layer was re-extracted with dichlormethane. The combined organics were dried over Na_2SO_4 and the solvent was removed under reduced pressure. The resultant oil product was purified by flash column chromatography using pentane/ethyl acetate (5:1 + 0.1% triethylamine) to obtain desired compound **11** as a clear oil.

Yield: 55% (1.77g). $\mathbf{R_f}$ 0.30 (pentane/ethyl acetate, 5:1);

^{1}H-NMR (300 MHz, $CDCl_3$): δ = 1.09-1.21 (m, 12H, (^{i}Pr)$_2$), 1.32 (d, $^3J_{H-H}$ = 7.1 Hz, 3H, CH_3), 2.32 (td, $^3J_{H-H}$ = 6.5 Hz, 0.7 Hz, 1 H, CH), 2.48 (t, $^3J_{H-H}$ = 6.3 Hz, 2H, CH_2), 2.61 (t, $^3J_{H-H}$ = 6.3 Hz, 2H, CH_2CN), 2.66-2.72 (m, 2H, CH_2CN), 3.45-3.65 (m, 5H, CH_2, 3 x CH), 3.79 (s, 6H, 2 x OCH_3), 3.93-3.96 (m, 2H, OCH_2), 6.80-6.85 (m, 4H, H_{Ar}), 7.18-7,30 (m, 7H, H_{Ar}), 7.42-7.48 (m, 2H, H_{Ar}) ppm. **^{31}P-NMR** (75 MHz, $CDCl_3$, RT): δ = 148.2, 148.9 ppm. **^{13}C-NMR** (125 MHz, $CDCl_3$, RT): δ = 18.8 (1C, CH_3), 18.9 (1C, CH_2), 20.2 (1C, CH_2), 24.2 (4C, 4 x CH_3), 26.7 (1C, CH_2), 27.0 (1C, CH_2), 42.9 (2C, 2 x CH), 43.4 (1C, CH), 55.2 (2C, C_{DMT}, 2 x OCH_3), 57.9 (1C, CH_2), 59.3 (1C, CH_2), 63.4 (1C, CH), 85.5 (1C, C_{DMT}), 113.1 (4C, 4 x CH, C_{DMT}), 117.8 (1C, CN), 118.4 (1C, CN), 126.9, 127.8, 128.2, 130.2 (9C, 9 x CH, C_{DMT}), 136.0 (2C, C_{DMT}), 144.8 (1C, C_{DMT}), 158.5 (2C, C_{DMT}) ppm.

ESI-MS *m/z* = 700.2 $[M+Na]^+$. **HRMS (ESI)**: calcd *m/z* = 700.2944 for $C_{37}H_{48}N_3O_5PSNa$, found 700.2948.

Synthesis and analytical data of oligonucleotides ON1 and ON2

The synthesis of oligonucleotides was carried out on a K&A H-8 DNA/RNA synthesizer using standard β-cyanoethyl phosphoramidite method on 0.2 µmol scale applying commercially available 2-cyanoethylphosphoramidites. Anhydrous acetonitrile was used as solvent. Reagents and CPGs were purchased from EMP BIOTECH and Sigma-Aldrich. Complementary template strands were purchased from BIOMERS. A standard method to incorporate the modified 2-cyanoethylphosphoramidites was used with the extended coupling time of 3 min for modified building blocks. The oligonucleotides were synthesized in DMT-ON mode and cleaved from the solid support with concentrated ammonia at 55 °C for 12 h. The obtained suspension was filtered and the filtrate was lyophilized. The resultant residue of tritylated oligonucleotides was first purified by reverse phase HPLC on a JASCO system equipped with two pumps PU-2080Plus, a diode array multi wavelength detector MD2010Plus a 3-line degasser DG-208053. A semipreparative C18 coloumn (VP 250/10

Nucleodur 100-5 C18ec) with a flow rate 3 mL/min was used. As elution buffer B1 [MeCN/TEAA (0.1 M, pH 7), 70:30] in A1 (0.1 M TEAA buffer pH 7) was used. UV absorption for oligonucleotides was detected at 260 nm. The fractions containing desired product were collected and lyophilized. For the detritylation of the oligonucleotides, 80% acetic acid was used over a period of 1 h. Prior to ESI-MS, the oligonucleotides were desalted using Sep-Pak C18 cartridges following the standard desalting protocol. The oligonucleotides were lyophilized and quantified by their absorbance in H_2O at 260 nm on a NanoDrop 2000c UV–vis spectrometer.

Table S1: Retention times (HPLC) and MS data of synthesized oligonucleotides **ON1** and **ON2**.

**Strand**	**Sequence (5'-3')**	**Retention time t_R**	**Calculated *m*/*z***	**Found *m*/*z***
**ON1**	CGCTAT**X**TATCGC[a]	19 min[c]	3763.49	3761.67
**ON2**	GCGATA**Y**ATAGCG[b]	21 min[d]	3929.69	3929.69

[a]'**X**' represents the threoninol site with deprotected amine group. [b]'**Y**' represents the threoninol site with cyanoethyl protected thiol group. [c]Gradient 5–30% B1 over 30 min. [d]Gradient 5–30% B1 over 30 min.

Experimental procedures for dynamic imine exchange reactions

Prior to the reactions, stock solutions of **ON1**, template strands and the nucleobase aldehydes were prepared freshly by dissolving the strands in 20 mM sodium phosphate buffer (1 M NaCl pH 6). **ON1** (50 L of a 200 µM stock solution, 10.0 nmol, 1.0 equiv), template strand (50.0 µL of a 200 µM stock solution, 10.0 nmol, 1.0 equiv), nucleobase-aldehydes $\mathbf{G_{CHO}}$, $\mathbf{C_{CHO}}$, $\mathbf{A_{CHO}}$, $\mathbf{T_{CHO}}$ (each in 40 µL of a 7 mM stock solution, 280 nmol, 28 equiv) were mixed and incubated at room temperature for 3 h. For reduction the $NaBH_3CN$ (50 µL of 1 M stock solution) was added and shaking continued for 1 h. The reaction mixture was purified by gel filtration (Centri·Pure N10 columns) to remove excess of aldehydes and salts. After lyophilization of the samples, crude products were analyzed using DNAPac PA200 4x5 mm, *DIONEX* Ion exchange column with a flow rate 1 mL/min. As elution buffer two different solvent systems were used: B2 (20 mM Tris-Cl, 1.25 M NaCl, pH 8.0) in A2 (20 mM Tris-Cl, pH 8.0) at a temperature of 80 °C or B3 (10 mM NaOH, 1.25 M NaCl, pH 12) in A3 (10 mM NaOH, pH 12) at room temperature.

Analytical data of products in dynamic library

The Watson–Crick coplementary base-pair products were obtained in presence of one single nucleobase aldehyde in four individual reactions as described above. To each of these reactions, an appropriate template strand ($\mathbf{T_C}$, $\mathbf{T_G}$, $\mathbf{T_T}$ or $\mathbf{T_A}$) was added, the one which has the complementary base of the added nucleobase-aldehyde**.** The retention times of the starting compounds and the reaction produts are reported in Table S2.

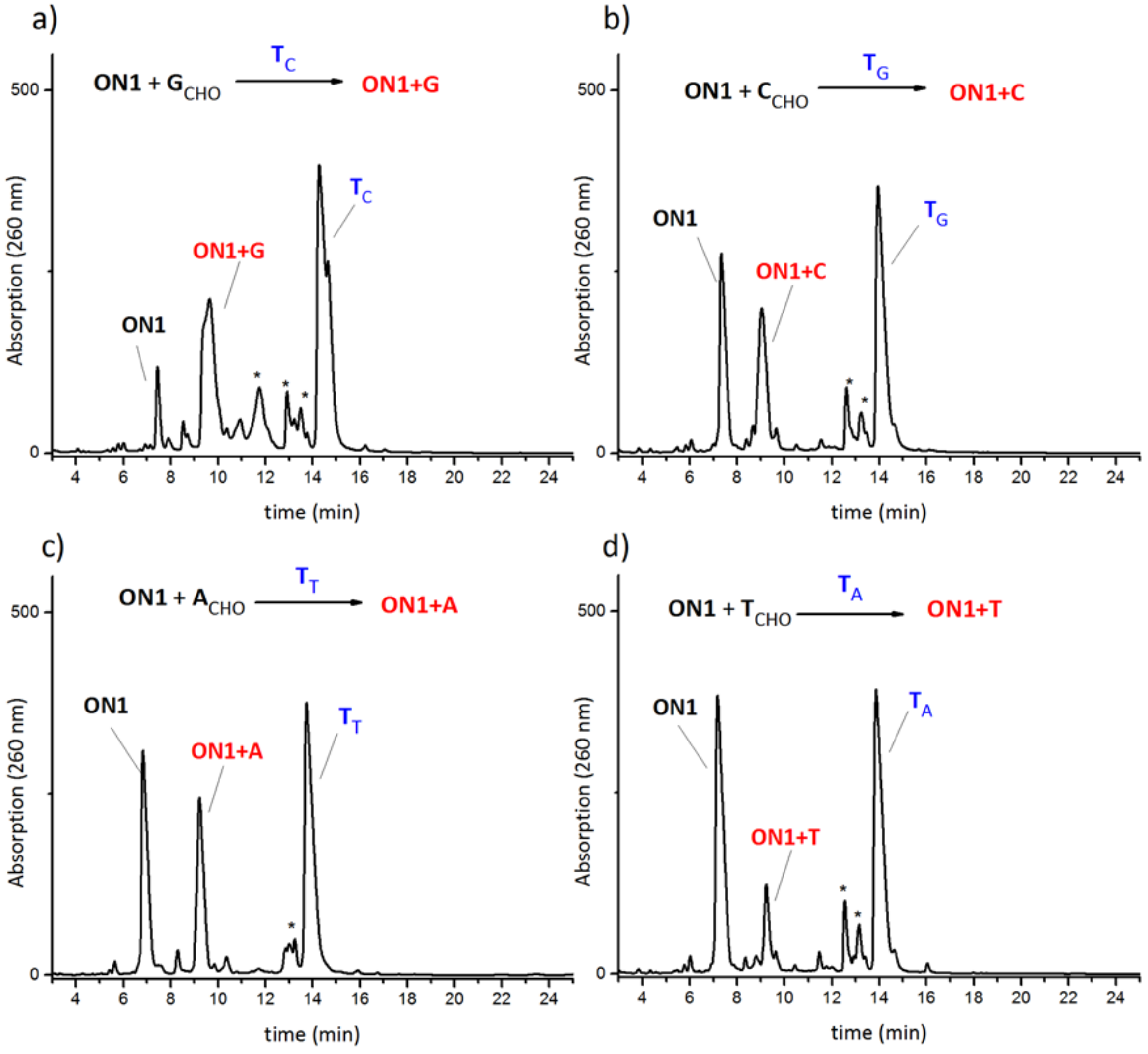

Figure S1: Representative AE-HPLC profiles after reductive amination between **ON1** and one of the nucleobases and one of the DNA-template: a) $\mathbf{G_{CHO}}$ and $\mathbf{T_C}$; b) $\mathbf{C_{CHO}}$ and $\mathbf{T_G}$; c) $\mathbf{A_{CHO}}$ and $\mathbf{T_T}$; d) $\mathbf{T_{CHO}}$ and $\mathbf{T_A}$. Solvent system: A2 (20 mM Tris-Cl, pH 8), B2 (20 mM Tris-Cl, 1.25 M NaCl, pH 8). Gradient 25–60% B2. Each HPLC run was performed at 80 °C. Black stars represent inpurities derived commercial oligonucleotide samples.

Table S2: Retention times (AE-HPLC) and mass spectral data of **ON1** and product strads after reductive amination.

**Strand**	**Sequence (5'-3')**	t_R	**Calculated *m/z***	**Found *m/z***
**ON1**	CGCTAT**X**TATCGC	7.3 min	3763.49	3761.67
**ON1+G**	CGCTAT$\mathbf{X^G}$TATCGC	9.6 min	3940.66	3938.73
**ON1+C**	CGCTAT$\mathbf{X^C}$TATCGC	9.0 min	3900.63	3898.73
**ON1+A**	CGCTAT$\mathbf{X^A}$TATCGC	9.2 min	3924.66	3922.74
**ON1+T**	CGCTAT$\mathbf{X^T}$TATCGC	9.3 min	3915.64	3913.72

Table S3: Ratio of AE-HPLC peak areas resulted from reductive amination between **ON1** and one of the nucleobases.

**Figure S1**	**Reaction**	**Template**	**Product**	**Product: ON1**[a]
a	**ON1**+$\mathbf{G}_{CHO}$	C	**ON1+G**	6:1
b	**ON1**+$\mathbf{C}_{CHO}$	G	**ON1+C**	1:1
c	**ON1**+$\mathbf{A}_{CHO}$	T	**ON1+A**	1:1
d	**ON1**+$\mathbf{T}_{CHO}$	A	**ON1+T**	1:4

[a] The conversion of **ON1** was determined by a comparison of the peak areas of the product and the starting material.

Condition of HPLC measurments in exepriments presented in Figure 4.

Solvent system: A2 (20 mM Tris-Cl, pH 8), B2 (20 mM Tris-Cl, 1.25 M NaCl, pH 8). Gradient 25–60% B2. Each HPLC run was performed at 80 °C**.**

Condition of HPLC measurments in exepriments presented in Figure 5.

Solvent system: A3 (10 mM NaOH, pH 12), B3 (10 mM NaOH, 1.25 M NaCl, pH 12). Gradient 35–70% B3. Each HPLC run was performed at room temperature.

References

[1] H. Asanuma, Y. Hara, A. Noguchi, K. Sano, H. Kashida, *Tetrahedron Lett.* **2008**, *49*, 5144–5146.

[2] H. Asanuma, T. Toda, K. Murayama, X. Liang, H. Kashida, *J. Am. Chem. Soc.* **2010**, *132*, 14702–14703.

[3] D. E. Bierer, J. M. Dener, L. G. Dubenko, R. E. Gerber, J. Litvak, S. Peterli, P. Peterli-Roth, T. V. Truong, G. Mao, B. E. Bauer, *J. Med. Chem.* **1995**, *38*, 2628–2648.

[4] Y. Shimohigashi, *Bull. Chem. Soc. Jpn.* **1979**, *52*, 949–950.
